# Nasally administered *Lactobacillus rhamnosus* strains differentially modulate respiratory antiviral immune responses and induce protection against respiratory syncytial virus infection

**DOI:** 10.1186/1471-2172-14-40

**Published:** 2013-08-15

**Authors:** Yohsuke Tomosada, Eriko Chiba, Hortensia Zelaya, Takuya Takahashi, Kohichiro Tsukida, Haruki Kitazawa, Susana Alvarez, Julio Villena

**Affiliations:** 1 Food and Feed Immunology Group, Graduate School of Agricultural Science, Tohoku University, Sendai, Japan; 2 Immunobiotics Research Group, Laboratory of Clinical and Experimental Biochemistry, Reference Centre for Lactobacilli (CERELA-CONICET), Tucuman, Argentina; 3 Applied Biochemistry Institute, Faculty of Biochemistry, Chemistry and Pharmacy, Tucuman University, Tucuman, Argentina

**Keywords:** *Lactobacillus rhamnosus*, Nasal treatment, Poly(I:C), Sntiviral immunity, Respiratory tract, Respiratory syncytial virus

## Abstract

**Background:**

Some studies have shown that nasally administered immunobiotics had the potential to improve the outcome of influenza virus infection. However, the capacity of immunobiotics to improve protection against respiratory syncytial virus (RSV) infection was not investigated before.

**Objective:**

The aims of this study were: a) to evaluate whether the nasal administration of *Lactobacillus rhamnosus* CRL1505 (Lr05) and *L. rhamnosus* CRL1506 (Lr06) are able to improve respiratory antiviral defenses and beneficially modulate the immune response triggered by TLR3/RIG-I activation; b) to investigate whether viability of Lr05 or Lr06 is indispensable to modulate respiratory immunity and; c) to evaluate the capacity of Lr05 and Lr06 to improve the resistance of infant mice against RSV infection.

**Results:**

Nasally administered Lr05 and Lr06 differentially modulated the TLR3/RIG-I-triggered antiviral respiratory immune response. Lr06 administration significantly modulated the production of IFN-α, IFN-β and IL-6 in the response to poly(I:C) challenge, while nasal priming with Lr05 was more effective to improve levels of IFN-γ and IL-10. Both viable Lr05 and Lr06 strains increased the resistance of infant mice to RSV infection while only heat-killed Lr05 showed a protective effect similar to those observed with viable strains.

**Conclusions:**

The present work demonstrated that nasal administration of immunobiotics is able to beneficially modulate the immune response triggered by TLR3/RIG-I activation in the respiratory tract and to increase the resistance of mice to the challenge with RSV. Comparative studies using two *Lactobacillus rhamnosus* strains of the same origin and with similar technological properties showed that each strain has an specific immunoregulatory effect in the respiratory tract and that they differentially modulate the immune response after poly(I:C) or RSV challenges, conferring different degree of protection and using distinct immune mechanisms. We also demonstrated in this work that it is possible to beneficially modulate the respiratory defenses against RSV by using heat-killed immunobiotics.

## Background

Acute lower respiratory tract infections are a persistent public health problem. Despite the remarkable advances in antibiotic therapies, diagnostic tools, prevention campaigns and intensive care, respiratory infections are still among the primary causes of death worldwide, and there have been no significant changes in mortality in the last decades [1]. Childhood acute community-acquired pneumonia is one of the leading causes of morbidity and mortality in developing countries. In children who have not received prior antibiotic therapy, the main bacterial causes of clinical pneumonia in developing countries are *Streptococcus pneumoniae* and *Haemophilus influenzae* type b, and the main viral cause is the respiratory syncytial virus [2].

Respiratory syncytial virus (RSV), a pneumovirus in the family *Paramyxoviridae*, infects nearly all children within the first 3 years of life. Primary RSV infections can cause severe bronchiolitis and pneumonia, which are associated with significantly increased risk of developing wheeze during childhood that lasts until teenage years. Symptomatic reinfections occur in every age group, but the frequency and severity of symptoms are highest in children below 5 years of age [3]. As described for several respiratory viruses, such as pandemic influenza virus strains and the human coronavirus that causes SARS, it is believed that the immune response plays a critical role in the outcome of RSV-induced bronchiolitis and pneumonia. Acute RSV infection is able to induce an exacerbated disease due to immune-mediated pulmonary injury resulting in severe morbidity and mortality [4]. Therefore, identifying novel approaches to modulate virus-induced immunopathology would be beneficial in treating acute RSV infections.

Several studies have demonstrated that certain lactic acid bacteria (LAB) strains can exert their beneficial effect on the host through their immunomudulatory activity. In this regard, some studies have centered on whether immunoregulatory probiotic LAB (immunobiotics) might sufficiently stimulate the common mucosal immune system to provide protection in other mucosal sites distant from the gut [5]. The studies of our laboratory demonstrated that some orally administered LAB are able to increase *S. pneumoniae* clearance rates in lung and blood, improve survival of infected mice and reduce lung injuries [5-8]. Moreover, we found that the effects of LAB treatments were related to an up-regulation of both respiratory innate and adaptive immune responses. In addition, considering that the nasal route can induce systemic and respiratory immune responses superior to those obtained using oral stimulation [9], we also focused on the ability of nasal stimulation with immunobiotics to improve respiratory immune responses. Our studies showed that nasally administered LAB are capable of modulating lung immunity and increase resistance against *S. pneumoniae* in both immunocompetent and immunocompromised mice and that in many cases, nasal priming is more effective than oral administration to beneficially modulate the respiratory immunity [10,11].

Recently, our laboratory studied the capacity of immunobiotics to improve respiratory antiviral immune response. To mimic the pro-inflammatory and physiopathological consequences of RNA viral infections in the lung such as those induced by RSV infection, we used an experimental model of lung inflammation based on the administration of the artificial toll-like receptor 3 (TLR3) and retinoic acid–inducible gene I (RIG-I) ligand and dsRNA analog poly(I:C) [12]. *In vivo* studies using mice demonstrated that nasally administered poly(I:C) results in TLR3- and CXCR2-dependent neutrophilic pulmonary inflammation, bronchiolar epithelial hypertrophy, interstitial edema and altered lung function [13,14]. These changes are accompanied by elevated levels of interleukin (IL)-8, RANTES, monocyte chemotactic protein (MIP)-1, and type I interferons (IFNs) in broncho-alveolar lavages (BAL) [13]. When we evaluated the effect of two Lactobacillus strains, *Lactobacillus rhamnosus* CRL1505 (Lr05) and *L. rhamnosus* CRL1506 (Lr06) in this mice model, we found that orally administered Lr05 beneficially regulate the balance between pro-inflammatory mediators and IL-10 in lung of poly(I:C)-challenged mice, allowing an effective control of the inflammatory response and avoiding tissue damage [12]. Moreover, our studies demonstrated that Lr05 is able to increase the number of CD3^+^CD4^+^IFN-γ^+^ T cells in the gut, induce the mobilization of these cells into the respiratory mucosa and improve the local production of IFN-γ and the activity of lung antigen presenting cells (APCs) [12]. Our results suggested that Lr05 is a potent inducer of antiviral cytokines and may be useful as a prophylactic agent to control respiratory virus infections. However, whether the nasal priming with Lr05 is more effective than oral administration to beneficially modulate the respiratory immune response triggered by poly(I:C) challenge has not been evaluated before. Moreover, further studies using real challenges with respiratory viruses such as RSV are needed in order to conclusively demonstrate the protective effect of Lr05.

Considering this background, the aims of this study were: a) to investigate whether the nasal administration of Lr05 or Lr06 are able to improve respiratory antiviral defenses and beneficially modulate the immune response triggered by TLR3/RIG-I activation; b) to evaluate whether viability of Lr05 or Lr05 is indispensable to modulate respiratory immunity considering that it was reported that heat-killed lactobacilli strains are able to improve lung defenses [11,15,16] and; c) to conclusively demonstrate the protective effect of Lr05 and Lr06 by evaluating their capacity to improve the resistance of infant mice against RSV challenge.

## Methods

### Microorganisms

*Lactobacillus rhamnosus* CRL1505 (Lr05) and CRL1506 (Lr06) were obtained from the CERELA culture collection (Chacabuco 145, San Miguel de Tucumán, Argentina). Both strains were selected because their previously reported immunomodulatory capacities [6,12]. The culture was kept freeze-dried and then rehydrated using the following medium: peptone 15.0 g, tryptone 10.0 g, meat extract 5.0 g, distilled water 1 l, pH 7. It was cultured for 12 h at 37°C (final log phase) in Man-Rogosa-Sharpe broth (MRS, Oxoid). The bacteria were harvested by centrifugation at 3000 g for 10 min, washed three times with sterile 0.01 mol/l phosphate buffer saline (PBS), pH 7.2, and resuspended in sterile 10% non-fat milk. Non-viable Lr05 and Lr06, designated as HkLr05 and HkLr06 respectively, were obtained as follows: bacteria were killed by tyndallization in a water bath at 80°C for 30 min and the lack of bacterial growth was confirmed using MRS agar plates [11].

### Animals and feeding procedures

Female 3-week-old BALB/c mice were obtained from the closed colony kept at Tohoku University. They were housed in plastic cages at room temperature. Mice were housed individually during the experiments and the assays for each parameter studied were performed in 5–6 mice per group for each time point. Lr05, Lr06, HkLr05 or HkLr06 were nasally administered to different groups of mice for 2 consecutive days at a dose of 10^8^ cells/mouse/day in 50 μl of PBS. The treated groups and the untreated control group were fed a conventional balanced diet *ad libitum*. This study was carried out in strict accordance with the recommendations in the Guide for the Care and Use of Laboratory Animals of the Guidelines for Animal Experimentation of Tohoku University, Sendai, Japan. The present study was approved by the Institution Animal Care and Use Committee of Tohoku University and all efforts were made to minimize suffering [5-8].

### Intranasal administration of poly(I:C)

Administration of the viral pathogen molecular pattern poly(I:C) was performed on day 3, after the two days treatments with lactobacilli, as described previously [12]. Mice were lightly anesthetized and 100 μl of PBS, containing 250 μg poly(I:C) (equivalent to 10 mg/kg body weight), was administered dropwise, via the nares. Control animals received 100 μl of PBS. Mice received three doses of poly(I:C) or PBS with 24 hs rest period between each administration.

### Cytokine concentrations in serum and broncho-alveolar lavages (BAL)

Blood samples were obtained through cardiac puncture at the end of each treatment and collected in heparinized tubes. BAL samples were obtained as described previously [8]. Briefly, the trachea was exposed and intubated with a catheter, and 2 sequential bronchoalveolar lavages were performed in each mouse by injecting sterile PBS; the recovered fluid was centrifuged for 10 min at 900 × g; the pellet was used to make smears that were stained for cell counts; and the fluid was frozen at −70°C for subsequent cytokines analyses. Tumour necrosis factor (TNF)-α, IFN-α, IFN-β, IFN-γ, IL-6 and IL-10 concentrations in serum and BAL were measured with commercially available enzyme-linked immunosorbent assay (ELISA) technique kits following the manufacturer's recommendations (R&D Systems, MN, USA) [6].

### Lung injury parameters

Protein and albumin content, a measure to quantitate increased permeability of the bronchoalveolar–capillarity barrier, and lactate dehydrogenase (LDH) activity, an indicator of general cytotoxicity, were determined in the acellular BAL fluid [8]. Protein content was measured by the bicinchoninic acid assay (BCA) protein assay (Pierce Biotechnology Inc., Rockford, IL). Albumin content was determined colorimetrically based on albumin binding to bromcresol green using an albumin diagnostic kit (Wiener Lab, Buenos Aires, Argentina). LDH activity, expressed as units per liter of BAL fluid, was determined by measuring the formation of the reduced form of nicotinamide adenine dinucleotide (NAD) using the Wiener reagents and procedures (Wiener Lab). Lung wet:dry weight ratio was measured as previously described [12]. Wet:dry weight ratio was calculated as an index of intrapulmonary fluid accumulation, without correction for blood content.

### Lung cells preparation

Single lung cells from mice were prepared using the previously described method [12]. Mice were anaesthetized with diethyl ether and killed the next day by exsanguination. Lungs were removed, finely minced and incubated for 90 min with 300 U of collagenase (Yakult Honsha Co., Tokyo, Japan) in 15 ml of RPMI 1640 medium (Sigma, Tokyo, Japan). To dissociate the tissue into single cells, collagenase-treated minced lungs were gently tapped into a plastic dish. After removal of debris, erythrocytes were depleted by hypotonic lysis. The cells were washed with RPMI medium supplemented with 100 U/ml of penicillin and 100 mg/ml of streptomycin and then resuspended in a medium supplemented with 10% heat-inactivated foetal calf serum (FCS). Cells were counted using Trypan Blue exclusion and then resuspended at an appropriate concentration of 5 × 10^6^ cells/ml.

### Flow cytometry studies

Lung cell suspensions were pre-incubated with anti-mouse CD32/CD16 monoclonal antibody (Fc block) for 15 min at 4°C. Cells were incubated in the antibody mixes for 30 min at 4°C and washed with FACS buffer. The following antibodies from BD PharMingen were used: anti-mouse CD3-FITC, anti-mouse CD4-PE, anti-mouse CD8-PE, anti-mouse IFN-γ-APC, anti-mouse CD11b-FITC, anti-mouse CD11c-PE, anti-mouse IFN-γ-PE, anti-mouse MHC-II-PE, anti-mouse IL-10-PE and anti-mouse CD103-biotin. Following incubation with biotinylated primary antibodies, the labeling was revealed using streptavidin-PercP. In all cases, cells were then acquired on a BD FACSCalibur™ flow cytometer (BD Biosciences) and data were analyzed with FlowJo software (TreeStar). The total number of cells in each population was determined by multiplying the percentages of subsets within a series of marker negative or positive gates by the total cell number determined for each tissue [12,17].

### Virus and infection

Human RSV strain A2 was grown in Vero cells as described by Murawski et al. [18]. Briefly, Vero cells were infected with RSV at a multiplicity of infection (MOI) of 1 in 5 ml of Dulbecco’s modified Eagle’s medium (DMEM). Cells were infected for 2.5 h at 37°C and 5% CO_2_. After infection, 7 ml of DMEM with 10% fetal bovine serum (Sigma, Tokyo, Japan), 0.1% penicillin-streptomycin (Pen/Strep) (Sigma, Tokyo, Japan), and 0.001% ciprofloxacin (Bayer) was added to the flask. Flasks were incubated until extensive syncytium formation was observed. Then, cells were scraped from the flask and sonicated three times, 5 s per time, at 25 W on ice. Cell debris was removed by centrifugation at 700 g for 10 min at 4°C. Virus supernatant was sucrose density gradient purified and stored in 30% sucrose at −80°C. Uninfected flasks were treated identically to generate Vero cell lysate control. For *in vivo* infection, mice were lightly anesthetized with isoflurane and intranasally challenged with 2.4 × 10^6^ PFU of RSV strain A2.

### RSV immunoplaque assay

Lung tissue was removed without BAL harvest and stored in 30% sucrose for plaque assay. Lungs were homogenized using a pellet pestle and centrifuged at 2,600 × g for 10 min at 4°C to clarify supernatant. Twenty-four-well tissue culture plates were seeded with 1.5 × 10^5^ Vero cells/well in DMEM containing 10% FBS, 0.1% Pen/Strep, and 0.001% ciprofloxacin. Cells were incubated overnight at 37°C and 5% CO_2_. Medium was removed from confluent monolayers, and serial dilutions of lung tissue-clarified supernatants were absorbed to monolayers. All samples were run in triplicate wells. Plates were incubated at 37°C and 5% CO^2^ for 2.5 h for optimum infection. After incubation, supernatant was removed, and 1 ml of fresh DMEM medium containing containing 10% FBS, 0.1% Pen/Strep, and 0.001% ciprofloxacin was overlaid on monolayers. When extensive syncytia developed, the overlay was removed and monolayers were fixed with 1 ml of ice-cold acetone:methanol (60:40). Primary RSV anti-F (clones 131-2A; Chemicon) and anti-G (Mouse monoclonal [8C5 (9B6)] to RSV glycoprotein, Abcam) antibodies were added to wells for 2 h, followed by secondary horseradish peroxidase anti-mouse immunoglobulin antibody (Anti-mouse IgG, HRP-linked Antibody #7076, Cell signaling Tehcnology) for 1 h. Plates washed twice with PBS containing 0.5% Tween 20 (Sigma) after each antibody incubation step. Individual plaques were developed using a DAB substrate kit (ab64238, Abcam) following manufacture’s specifications. Results for immunoplaque assay were expressed as log10 PFU/g of lung.

### Statistical analysis

Experiments were performed in triplicate and results were expressed as mean ± standard deviation (SD). After verification of the normal distribution of data, 2-way ANOVA was used. Tukey's test (for pairwise comparisons of the means) was used to test for differences between the groups. Differences were considered significant at p<0.05.

## Results

### Nasally administered L. rhamnosus CRL1505 and L. rhamnosus CRL1506 differentially modulate respiratory immunity

**Figure 1:**
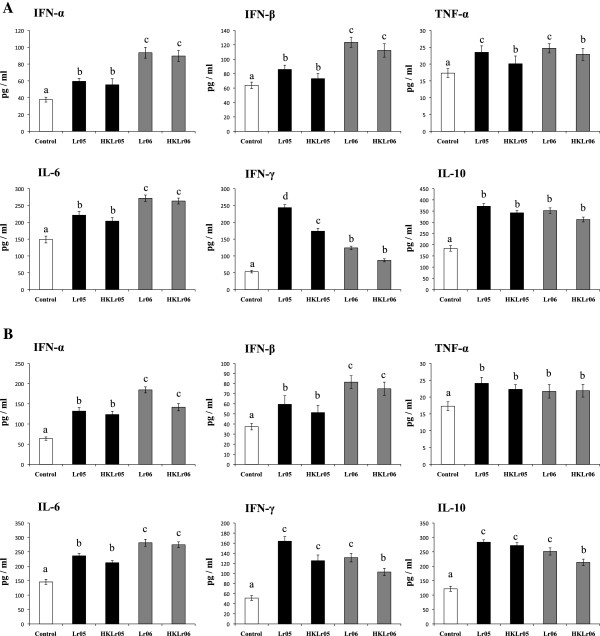
**Effect of lactobacilli on systemic and respiratory immunity.** Effect of viable (Lr05) or heat-killed (HkLr05) *Lactobacillus rhamnosus* CRL1505 and viable (Lr06) or heat-killed (HkLr06) *L. rhamnosus* CRL1506 nasal administration on the tumor necrosis factor (TNF)-α, interferon (IFN)-α, IFN-β, IFN-γ, interleukin (IL)-6, and IL-10 concentrations in broncho-alveolar lavages **(A)** and serum **(B)**. Lr05, Lr06, HkLr05 or HkLr06 were nasally administered to different groups of mice for 2 consecutive days at a dose of 10^8^ cells/mouse/day and the levels of BAL and serum cytokines were studied on day 3. The results represent data from three independent experiments. Different letters indicate significant differences (P < 0.05).

In order to evaluate the changes induced by nasally administered LAB in the respiratory immune system we determined the levels of different cytokines in BAL (Figure 1A). The four nasal treatments used in this study Lr05, HkLr05, Lr06 and HkLr06 increased the levels of IL-6, IFN-α and IFN-β in BAL, however concentrations of these cytokines were significantly higher in Lr06- and HkLr06-treated mice than in mice receiving Lr05 or HkLr05 (Figure 1A). All the treatments were also able to increase BAL IFN-γ, being both viable and heat-killed *L. rhamnosus* CRL1505 more efficient to improve the levels of this cytokine than *L. rhamnosus* CRL1506 (Figure 1A). BAL TNF-α and IL-10 concentrations were increased by the immunobiotic nasal treatments. We observed that viable cells were more efficient to upregulate TNF-α than heat-killed strains while no differences were found in IL-10 levels when comparing viable and heat-killed lactobacilli (Figure 1A). When we evaluated the levels of IL-6, IFN-α, IFN-β, IFN-γ, TNF-α and IL-10 in serum we found that lactobacilli treatments induced similar changes than those observed in the respiratory tract (Figure 1B). IL-6, IFN-α and IFN-β were more efficiently increased with *L. rhamnosus* CRL1506 treatments while the highest levels of serum IFN-γ were observed in Lr05 and HkLr05 groups (Figure 1B). Serum TNF-α and IL-10 concentrations were also enhanced by the immunobiotic nasal treatments, being viable and heat-killed lactobacilli equally effective to improve both cytokines whit the exception of HkLr06 that induced significantly lower levels of IL-10 when compared with the other treatments (Figure 1B).

**Figure 2:**
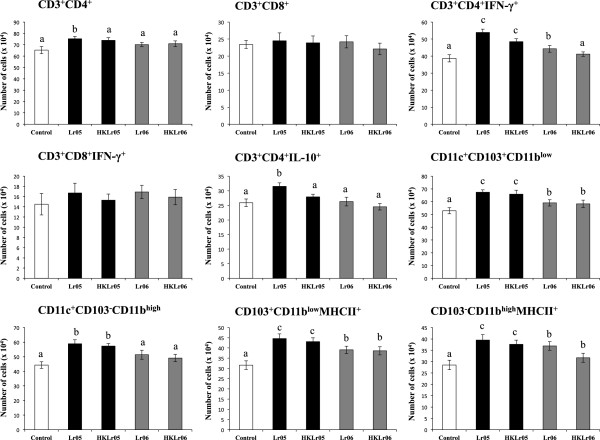
**Effect of lactobacilli on respiratory immune cells populations.** Effect of viable (Lr05) or heat-killed (HkLr05) *Lactobacillus rhamnosus* CRL1505 and viable (Lr06) or heat-killed (HkLr06) *L. rhamnosus* CRL1506 nasal administration on CD3+CD8+IFN-γ+, CD3+CD4+IFN-γ+ and CD3+CD4+IL-10+ T cells and CD11c+CD11b^low^CD103+ and CD11c+CD11b^high^CD103- dendritic cells from lung. Lr05, Lr06, HkLr05 or HkLr06 were nasally administered to different groups of mice for 2 consecutive days at a dose of 10^8^ cells/mouse/day and lung immune cells were studied on day 3. The results represent data from three independent experiments. Different letters indicate significant differences (P < 0.05).

We also evaluated the changes induced by nasally administered lactobacilli in lung immune cells using flow cytometry. Slightly increases of lung CD3^+^CD4^+^ T cells were observed in lactobacilli-treated mice, however when we studied the cells able to produce IFN-γ within this population, mice receiving Lr05, HkLr05 and Lr06 showed significant differences when compared to control mice (Figure 2). Nasally administered Lr05 and HkLr05 increased the number of CD3^+^CD4^+^IFN-γ^+^ T cells in lungs being Lr05 more efficient than HkLr05 to increase this cell population (Figure 2). Only Lr05 enhanced the number of CD3^+^CD4^+^IL-10^+^ T cells in lungs (Figure 2). No modifications were observed in the number of CD3^+^CD8^+^ and CD3^+^CD8^+^IFN-γ^+^ T cells in lactobacilli-treated mice (Figure 2).

Two populations of myeloid DCs can be defined in lungs using CD11c, CD11b, CD103 and MHC-II antibodies as described previously [12,19]: MHC-II^+^CD11c^+^CD11b^low^CD103^+^ and MHC-II^+^CD11c^+^CD11b^high^CD103^-^ cells. Therefore, we next aimed to evaluate the effect of nasally administered lactobacilli on these populations of DCs from lungs. Lr05 and HkLr05 significantly increased the number of both lung CD11c^+^CD11b^low^CD103^+^ and CD11c^+^CD11b^high^CD103^-^ DCs. In addition, Lr06 and HkLr06 enhanced the number of lung CD11c^+^CD11b^low^CD103^+^ DCs while no quantitative changes were detected in CD11c^+^CD11b^high^CD103^-^ DCs populations in lungs of Lr06- and HkLr06-treated mice (Figure 2). In addition, the expression of MHC-II in both DCs population was significantly improved with all the treatments, however *L. rhamnosus* CRL1505 was more efficient than *L. rhamnosus* CRL1506 to upregulate the expression of MHC-II in lung DCs (Figure 2).

### Poly(I:C)-induced lung injuries are reduced by nasally administered lactobacilli

**Figure 3:**
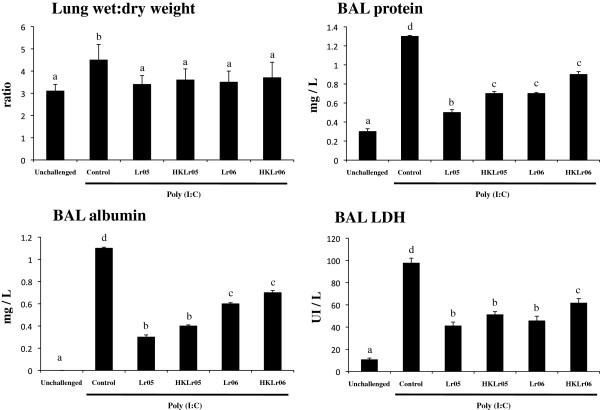
**Effect of lactobacilli on lung injuries induced by the nasal administration of the viral pathogen-associated molecular pattern poly(I:C).** Effect of viable (Lr05) or heat-killed (HkLr05) *Lactobacillus rhamnosus* CRL1505 and viable (Lr06) or heat-killed (HkLr06) *L. rhamnosus* CRL1506 nasal administration on lung wet:dry weight ratio, lactate dehydrogenase (LDH) activity and, albumin and protein concentrations in broncho-alveolar lavages (BAL) after the challenge with poly(I:C). Lr05, Lr06, HkLr05 or HkLr06 were nasally administered to different groups of mice for 2 consecutive days at a dose of 10^8^ cells/mouse/day. After lactobacilli treatment, mice received three doses of poly(I:C) with 24 hours rest period between each administration. Lung tissue injury markers were studied 24 hours after the third challenge with poly(I:C). The results represent data from three independent experiments. Different letters indicate significant differences (P < 0.05).

We next aimed to evaluate the effect of nasally administered lactobacilli on the immune response triggered by nasal administration of the viral pathogen-associated molecular pattern poly(I:C). Our previous work demonstrated that the nasal challenge of mice with poly(I:C) significantly alters lungs function and induce lung injuries [12]. In this experimental model an altered wet:dry weight ratio can be observed after poly(I:C) challenge (Figure 3). Moreover, significantly increased levels of LDH activity as well as protein and albumin concentrations can be found in BAL samples of challenged mice indicating that poly(I:C) produces local cellular damage and impairment of the alveolar-capillary barrier (Figure 3). We observed that nasally administered lactobacilli prior to poly(I:C) challenge significantly reduced wet:dry weight ratio and BAL LDH (Figure 3). In addition, all the treatments were able to significantly reduce BAL protein and albumin concentrations however *L. rhamnosus* CRL1505 was more efficient than the CRL1506 strain to reduce the impairment of the alveolar-capillary barrier (Figure 3).

### Nasally administered lactobacilli beneficially modulate immune response triggered by poly(I:C) challenge

**Figure 4:**
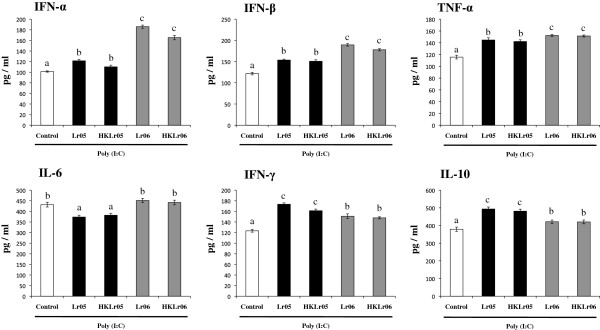
**Effect of lactobacilli on the production of cytokines induced by the nasal administration of the viral pathogen-associated molecular pattern poly(I:C).** Effect of viable (Lr05) or heat-killed (HkLr05) *Lactobacillus rhamnosus* CRL1505 and viable (Lr06) or heat-killed (HkLr06) *L. rhamnosus* CRL1506 nasal administration on the tumor necrosis factor (TNF)-α, interferon (IFN)-α, IFN-β, IFN-γ, interleukin (IL)-6 and IL-10 concentrations in broncho-alveolar lavages (BAL). Lr05, Lr06, HkLr05 or HkLr06 were nasally administered to different groups of mice for 2 consecutive days at a dose of 10^8^ cells/mouse/day. After lactobacilli treatment, mice received three doses of poly(I:C) with 24 hours rest period between each administration. BAL cytokines were studied 12 (TNF-α, IFN-α, IFN-β and IL-6) or 48 (IFN-γ and IL-10) hours after the third challenge with poly(I:C). The results represent data from three independent experiments. Different letters indicate significant differences (P < 0.05).

The pulmonary immune response induced by the nasal challenge with poly(I:C) and the effect of nasally administered lactobacilli in that response were next evaluated. We have previously study the levels and kinetics of IFN-α, IFN-β, IFN-γ, IL-6, IL-4, TNF-α, IL-1β, IL-8, MCP-1, IL-10 and TGF-β in BAL after poly(I:C) challenge and the effect of orally administered immunobiotics in that response [12]. The most significant changes induced by oral treatment with immunobiotics were found in the levels of IFN-α, IFN-β, IFN-γ, IL-6, TNF-α and IL-10, therefore these cytokines were studied in this work. Nasal administration of poly(I:C) significantly increased respiratory levels of the pro-inflammatory mediators IFN-α, IFN-β, IL-6 and TNF-α (Figure 4) as previously reported [12]. All lactobacilli treatments significantly increased the levels of BAL IFN-α, IFN-β and TNF-α however, Lr06 and HkLr06 were more efficient to enhance the concentration of these cytokines than Lr05 or HkLr05 (Figure 4). In addition, IL-6 was not modified in Lr06 and HkLr06 groups while the levels of this cytokine were lower in *L. rhamnosus* CRL1505-treated mice when compared to controls (Figure 4). Poly(I:C) challenge also induced an increase in the respiratory levels of IL-10 and IFN-γ and the levels of both cytokines were significantly higher in lactobacilli-treated mice, being Lr05 and HkLr05 more efficient than Lr06 and HkLr06 to achieve that effects (Figure 4). The nasal challenge with poly(I:C) also increased cytokines levels in serum as previously reported [12]. Moreover, the effect of Lr05, HkLr05, Lr06 and HkLr06 treatments on the production of serum IFN-α, IFN-β, IFN-γ, IL-6, TNF-α and IL-10 was similar to that found in BAL (data not shown).

**Figure 5:**
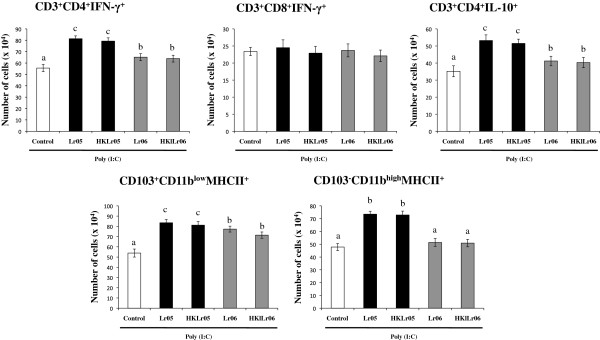
**Effect of lactobacilli on respiratory immune cells populations after the nasal administration of the viral pathogen-associated molecular pattern poly(I:C).** Effect of viable (Lr05) or heat-killed (HkLr05) *Lactobacillus rhamnosus* CRL1505 and viable (Lr06) or heat-killed (HkLr06) *L. rhamnosus* CRL1506 nasal administration on CD3+CD8+IFN-γ+, CD3+CD4+IFN-γ+ and CD3+CD4+IL-10+ T cells and CD11c+CD103+MHCII+ and CD11c+CD11b^high^MHCII+ dendritic cells from lung. Lr05, Lr06, HkLr05 or HkLr06 were nasally administered to different groups of mice for 2 consecutive days at a dose of 10^8^ cells/mouse/day. After lactobacilli treatment, mice received three doses of poly(I:C) with 24 hours rest period between each administration. Lung immune cells were studied 48 hours after the third challenge with poly(I:C). The results represent data from three independent experiments. Different letters indicate significant differences (P < 0.05).

We also studied the changes in lung immune cells induced by nasally administered lactobacilli in poly(I:C)-challenged mice (Figure 5). Poly(I:C) administration increased CD3^+^CD8^+^IFN-γ^+^ and CD3^+^CD4^+^IFN-γ^+^ T cells as we described previously [12]. In addition, in this work we also observed an increase in CD3^+^CD4^+^IL-10^+^ T cells after the challenge with poly(I:C) (Figure 5). Our results showed that nasally administered lactobacilli were able to increase both CD3^+^CD4^+^IFN-γ^+^ and CD3^+^CD4^+^IL-10^+^ T cells in lungs, however the levels of these cell populations in Lr05- and HkLr05-treated mice were significantly higher than those observed in Lr06 and HkLr06 groups (Figure 5). No differences were observed between lactobacilli-treated mice and controls when evaluating CD3^+^CD8^+^IFN-γ^+^ T cells (Figure 5). Poly(I:C) challenge also increased the number of pulmonary CD11b^high^CD103^-^MHC-II^+^ and CD11b^low^CD103^+^MHC-II^+^ DCs when compared to basal levels in all the experimental groups (Figure 5). Nasal administration of both *L. rhamnosus* CRL1505 and *L. rhamnosus* CRL1506 significantly increased the numbers of CD11b^low^CD103^+^MHC-II^+^ DCs cells in lungs when compared to controls while only Lr05- and HkLr05-treated mice showed improved levels of pulmonary CD11b^high^CD103^-^MHC-II^+^ DCs (Figure 5).

### Nasally administered lactobacilli improve resistance against respiratory syncytial virus infection

**Figure 6:**
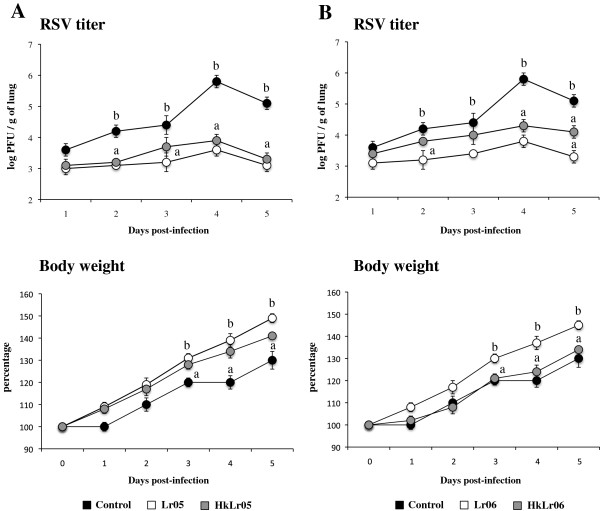
**Effect of lactobacilli on the resistance against respiratory syncytial virus (RSV) infection.** Effect of viable (Lr05) or heat-killed (HkLr05) *Lactobacillus rhamnosus* CRL1505 **(A)** and viable (Lr06) or heat-killed (HkLr06) *L. rhamnosus* CRL1506 **(B)** nasal administration on the resistance against RSV infection. Lr05, Lr06, HkLr05 or HkLr06 were nasally administered to different groups of mice for 2 consecutive days at a dose of 10^8^ cells/mouse/day. After lactobacilli treatment, mice were nasally challenged with RSV and lung virus titers and changes in body weight were studied on different time points after the challenge. The results represent data from three independent experiments. Different letters indicate significant differences (P < 0.05).

**Figure 7:**
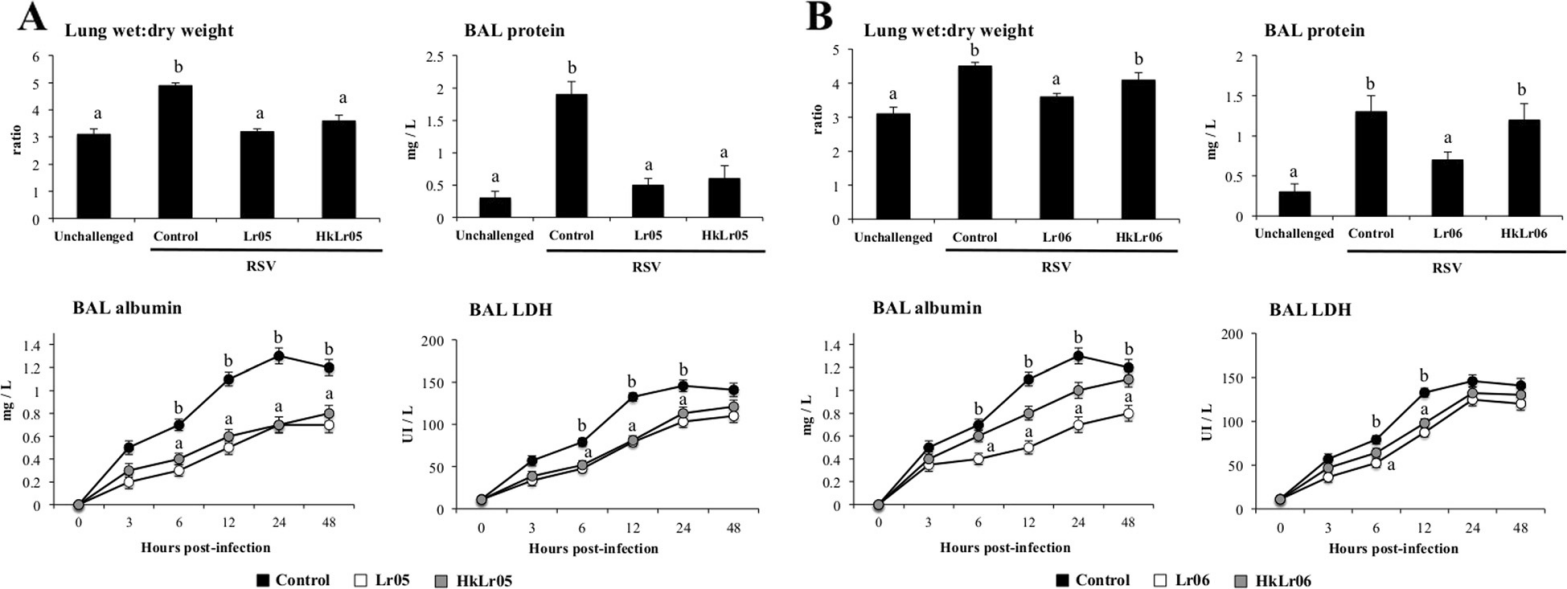
**Effect of lactobacilli on lung injuries induced by respiratory syncytial virus (RSV) infection.** Effect of viable (Lr05) or heat-killed (HkLr05) *Lactobacillus rhamnosus* CRL1505 **(A)** and viable (Lr06) or heat-killed (HkLr06) *L. rhamnosus* CRL1506 **(B)** nasal administration on lung tissue injuries induced by RSV infection. Lr05, Lr06, HkLr05 or HkLr06 were nasally administered to different groups of mice for 2 consecutive days at a dose of 10^8^ cells/mouse/day. After lactobacilli treatment, mice were nasally challenged with RSV and wet:dry weight ratio, lactate dehydrogenase (LDH) activity and, albumin and protein concentrations in broncho-alveolar lavages (BAL) were studied in different time points after the challenge. The results represent data from three independent experiments. Different letters indicate significant differences (P < 0.05).

We next addressed the question of whether changes observed in respiratory immune system caused by the intervention with immunobiotics affected the outcome of RSV infection in mice. Therefore, infant mice were nasally treated with Lr05, HkLr05, Lr06 or HkLr06 and then challenged with 10^6^ PFU of RSV. Viral loads in lungs of infected mice were followed for five days after the challenge (Figure 6). RSV was detected in lungs of all the experimental groups during the five days post-infection and all groups showed a peak of virus counts on day 4 after the challenge. However, lactobacilli-treated mice showed significantly lower lung viral loads when compared to control mice. Lr05 and HkLr05 treatments were equally effective to reduce RSV replication in lungs while in the case of *L. rhamnonus* CRL1506 viable bacteria was more effective than heat-killed cells to improve protection against the respiratory viral infection (Figure 6). In addition, we observed significantly differences in the body weight of infected mice when comparing lactobacilli-treated mice and controls (Figure 6). Lr05, HkLr05 and Lr06 significantly improved the body weight during RSV infection while HkLr06-tretated mice showed no differences when compared to controls (Figure 6). We also evaluated the markers of lung tissue damage in RSV-infected mice. As showed in Figure 7, challenge with RSV significantly increase lung wet:dry weight, BAL protein concentrations and LDH activity. All these markers of lung tissue damage were significantly higher in RSV-challenged control mice than in those previously treated with Lr05, HkLr05 or Lr06 (Figure 7). On the contrary, lung wet:dry weight, BAL protein concentrations and BAL LDH activity in HkLr06-treated mice were not different from controls (Figure 7).

### Nasally administered lactobacilli differentially modulate the cytokines response to respiratory syncytial virus infection

**Figure 8:**
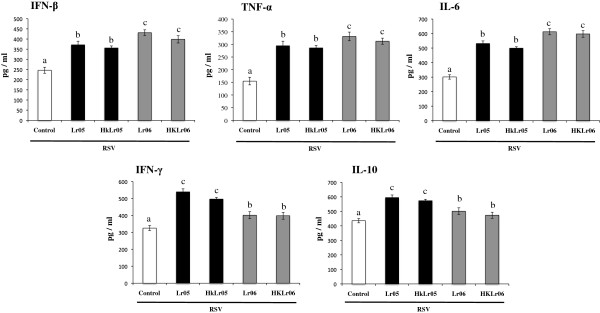
**Effect of lactobacilli on the production of cytokines induced by respiratory syncytial virus (RSV) infection.** Effect of viable (Lr05) or heat-killed (HkLr05) *Lactobacillus rhamnosus* CRL1505 and viable (Lr06) or heat-killed (HkLr06) *L. rhamnosus* CRL1506 nasal administration on the tumor necrosis factor (TNF)-α, interferon (IFN)-α, IFN-β, IFN-γ, interleukin (IL)-6 and IL-10 concentrations in broncho-alveolar lavages (BAL). Lr05, Lr06, HkLr05 or HkLr06 were nasally administered to different groups of mice for 2 consecutive days at a dose of 10^8^ cells/mouse/day. After lactobacilli treatment, mice were nasally challenged with RSV and BAL cytokines were studied 12 (TNF-α, IFN-β and IL-6) or 48 (IFN-γ and IL-10) hours after the challenge. The results represent data from three independent experiments. Different letters indicate significant differences (P < 0.05).

Finally, we addressed whether the improvement in the resistance against RSV induced by lactobacilli treatments was related to a differential modulation of cytokines production during infection (Figure 8). Therefore, we evaluate the levels of BAL and serum IL-6, IFN-β and TNF-α on day 2 post-infection and the concentrations of IFN-γ and IL-10 on day 5 after challenge. We observed that the challenge with RSV significantly increased the levels of the cytokines studied in all experimental groups. We also detected that lactobacilli-treated mice showed significantly higher levels of BAL IL-6, IFN-β and TNF-α than controls, being Lr06 and HkLr06 treatments more efficient than Lr05 or HkLr05 to improve the production of these factors (Figure 8). In addition, levels of IFN-γ and IL-10 were significantly improved by Lr05, HkLr05 and Lr06 treatments while nasal administration of HkLr06 increased IFN-γ but not IL-10 production in the respiratory tract. Moreover, Lr05 and HkLr05 were more effective than Lr06 to increase BAL IFN-γ and IL-10 concentrations in response to RSV challenge (Figure 8). The effect of Lr05, HkLr05, Lr06 and HkLr06 treatments on the production of serum TNF-α, IFN-β, IL-6, IFN-γ and IL-10 was similar to that found in BAL (data not shown).

## Discussion

In the present work we studied the effect of nasally administered immunobiotic lactobacilli on the respiratory antiviral immune response and evaluated their capacity to improve protection of infant mice against RSV infection. Three important conclusions can be inferred from the results presented in this study: a) nasally administered Lr05 and Lr06 differentially modulate the TLR3/RIG-I-triggered antiviral respiratory immune response; b) nasal priming with Lr05 or Lr06 strains increase the resistance of infant mice to RSV infection and; c) the viability of the immunobiotic strains is not a necessary condition to achieve the immunoregulatory protective effect.

*a) Nasally administered Lr05 and Lr06 differentially modulate the TLR3/RIG-I-triggered antiviral respiratory immune response.* We have previously demonstrated that nasal administration of three once-daily doses of poly(I:C) resulted in a marked impairment of lung function that was accompanied by inflammatory cell recruitment into the airways and the production of pro-inflammatory mediators [12]. Accordingly, in the present work we observed increased LDH activity and albumin concentration in BAL as well as increased levels of type I IFNs, TNF-α and IL-6, in the respiratory tract of poly(I:C)-challenged mice. We also showed that this immune response can be modulated with the preventive nasal administration of Lr05 or Lr06, demonstrating in addition that each strain has a different immunoregulatory effect. While Lr06 administration had a significant effect on the production of IFN-α, IFN-β and IL-6 in the response to poly(I:C) challenge, nasal priming with Lr05 was more effective to improve levels of IFN-γ and IL-10.

**Figure 9:**
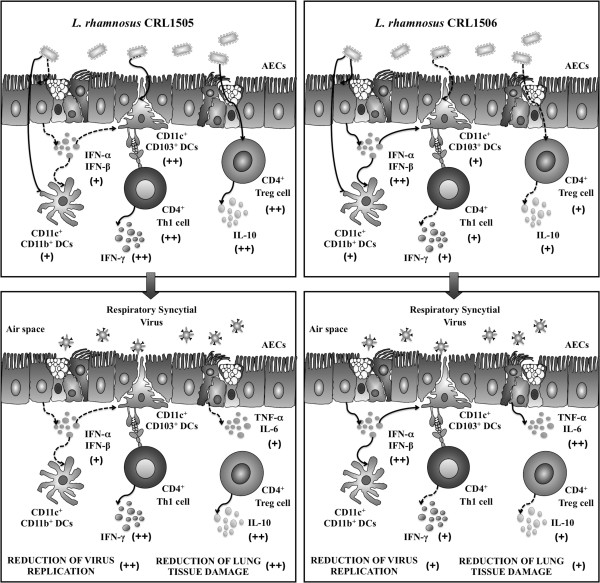
Proposed mechanism for the improvement of antiviral immunity and resistance against respiratory syncytial virus (RSV) infection induced by nasally administered lactobacilli.

It was demonstrated that poly(I:C) elicited the secretion of type I IFNs, TNF-α and IL-6 and other cytokines in respiratory epithelial cells [20], therefore a likely source of these cytokines following poly(I:C) administration may be the airway epithelium. Then, Lr06 administration would have a significant effect on respiratory epithelial cells. Taking into consideration that IFN-α and IFN-β up-regulate several genes involved in viral defense but also genes of major importance for the development of a strong Th1 response, it can be speculated that Lr06 may play an important role in the improvement of innate and specific immune responses against respiratory virus through the stimulation of antiviral defenses in epithelial cells (Figure 9). In addition, we have previously demonstrated that nasal administration of poly(I:C) activates respiratory MHC-II^+^CD11c^+^CD11b^low^CD103^+^ (CD103^+^ DCs) and MHC-II^+^CD11c^+^CD11b^high^CD103^-^ (CD11b^high^ DCs) cells and increases CD3^+^CD4^+^IFN-γ^+^ and CD3^+^CD4^+^IFN-γ^+^ T cells in lungs, indicating the generation of a Th1 response [12]. The result presented here shows that nasal administration of Lr05 has the capacity to improve Th1 response since higher levels of BAL IFN-γ and lung CD3^+^CD4^+^IFN-γ^+^ T cells were found in Lr05-treated mice. Moreover, we showed that Lr05 administration significantly activated CD103^+^ DCs, an affect that was not achieved by Lr06. Considering that recent studies suggested that lung CD103^+^ DCs are more potent at eliciting Th1 and Th17 responses than CD11b^high^ DCs [21], we can speculate that Lr05 is more efficient than Lr06 to stimulate CD103^+^ DCs and improve Th1 response in the respiratory tract (Figure 9).

Nasal treatment with lactobacilli significantly reduced lung injuries caused by poly(I:C) administration. We previously suggested that IL-10 would be valuable for attenuating inflammatory damage and pathophysiological alterations in lungs challenged with the viral pathogen-associated molecular pattern poly(:IC) [12]. We demonstrated here that both Lr05 and Lr06 significantly improved the production of IL-10 in response to poly(:IC), however Lr05 was more efficient than Lr06 to upregulate the levels of this cytokine in the respiratory tract. Moreover, the lung tissue injury markers used in this study were significantly lower in Lr05-treated mice than in those receiving the Lr06 strain. Therefore, we confirmed that there is a direct connection between the improvement of IL-10 levels induced by immunobiotics and the protection against lung injury after respiratory poly(I:C) challenge. Moreover, in this work we demonstrated that CD3^+^CD4^+^IL-10^+^ T cells would be the source of the IL-10 produced after poly(I:C) challenge and that this immune cell population would be quantitatively and functionally modulated by Lr05 since increased levels of CD3^+^CD4^+^IL-10^+^ T cells were found in lungs of Lr05-treated mice when compared with controls and those receiving Lr06 (Figure 9). Recently, Weiss et al. [4] demonstrated that CD4^+^ T cells produce the majority of IL-10 *in vivo* during an acute RSV infection and that this cell population is involved in the protective effect against lung tissue damage. Therefore, the increase in the numbers of CD4^+^IL-10^+^ T cells induced by nasal treatment with lactobacilli could have an important role in the protection against RSV infection.

Similar to the changes observed in intestinal immunity after oral administration of Lr05 or Lr06 [12], we demonstrated in this work that nasal priming with both lactobacilli has the ability to improve antiviral immunity but using different mechanisms (Figure 9). Moreover, considering that activating host immune responses during RSV infection is dependent on complex signaling events initiated in part by PRRs such as TLR3 or RIG-I and that these coordinated signaling events promote the production of cytokines, chemokines, IFN-α, IFN-β and IFN-γ in the lung that are crucial for the virus clearance, we speculated that Lr05 or Lr06 nasal treatments would beneficially modulate the immune response against RSV and improve resistance of mice against this viral respiratory infection.

*b) Nasal priming with Lr05 or Lr06 strains increase resistance of infant mice to RSV challenge.* In the last years, some lines of evidence showed that nasal administration of immunobiotics is able to increase resistance against respiratory viral infections [22]. It was reported that intranasal priming with *L. rhamnosus* GG to BALB/c mice significantly reduced the frequency of accumulated symptoms and induced a higher survival rate than control mice after challenge with influenza virus H1N1 [23]. Authors demonstrated that *L. rhamnosus* GG significantly increased lung NK cell activation and expression of TNF-α, IL-1β and MCP-1 enhancing respiratory cell-mediated immune responses. In addition, it was reported recently that nasal priming with lactobacilli is highly effective at suppressing virus-induced inflammation in a pneumonia virus mouse model [16]. Nasal priming with lactobacilli resulted in marked suppression of IFN-inducible protein-10 (CXCL10), MCP-1 (CCL2), neutrophil-activating protein-3 (CXCL1), MIP-1γ (CCL9), TNF, and eotaxin-2 (CCL24) in response to pneumovirus infection and significantly increased the resistance against the lethal disease. In this work we extend these findings by demonstrating that nasally administered immunobiotics are able to increase protection against RSV infection in infant mice.

We observed that challenge of three weeks old BALB/c mice with RSV significantly altered lung function, induced tissue injury and triggered inflammatory response. This is in line with previous work reporting that RSV intranasal infection led to significant clinical characteristics in female BALB/c mice [24]. Authors described ruffled fur and ataxia that occurred early after hour 12 post-RSV infection and continued for 3 days [24]. Natural human RSV infection in children and experimental RSV inoculation in mice result in prominent local secretion of pro-inflammatory cytokines, such as TNF-α, IL-6, IL-8, MIP-1, RANTES, and MCP-1 as well as type I IFNs [25]. It has been shown that type I IFNs, IL-6 and TNF-α contribute to clearance of the virus during the early stages of RSV infection however, continued production of these pro-inflammatory mediators exacerbates illness and tissue injuries during the late stages of RSV infection [26]. Interestingly, it was found that IL-10 deficiency during RSV challenge did not affect viral load, but led to markedly increased disease severity with enhanced weight loss, delayed recovery and a greater influx of inflammatory cells into the lung and airways and enhanced release of inflammatory mediators [27]. Therefore, during acute RSV infection, it is imperative that the host’s inflammatory response is tightly regulated, enabling virus elimination but limiting the detrimental effects of inflammation on the lung tissue. Then, an adequate balance of pro-inflammatory and anti-inflammatory factors is essential for a safe and effective antiviral immune response [28]. Moreover, considering that it was reported that no changes in peak viral titers or viral clearance were observed between IL-10 KO or anti–IL-10R mAb-treated mice compared with their controls [4], preventive or therapeutic approaches aimed at increasing IL-10 production may offer a means to decrease RSV-induced immunopathology without affecting viral clearance.

We demonstrated in this work that nasal administration of Lr05 or Lr06 improved the production of pro-inflammatory mediators in response to RSV challenge and also the production of IL-10, which would allow an effective immunological clearance of the virus without affecting lung tissue. Similarly to our results using poly(I:C) challenge, we observed that Lr05 was the strain with the highest capacity to improve levels of IL-10 and was more effective than Lr06 to enhance virus clearance and to protect lungs against the inflammatory damage. Then, our results also support the idea that modulation of respiratory IL-10 during RSV infection is an effective way to improve the outcome of viral disease. Moreover, we demonstrated here that nasally administered immunobiotics are an interesting alternative to achieve that immunoprotective effect.

Following RSV infection, there is an initial influx of NK cells to the site of infection that produce IFN-γ and are cytotoxic to virus-infected cells. This is followed by recruitment of helper CD4^+^ and cytotoxic CD8^+^ lymphocytes to the site of infection. IFN-γ enhances the differentiation of CD8^+^ lymphocytes and influences the differentiation of CD4^+^ lymphocytes that contribute to the generation and amplification of the humoral and cellular immune responses. While the RSV-specific T cell response plays a major role in viral clearance and the clinical outcome of infection, both Th2-biased CD4^+^ and CD8^+^ T cells have been implicated in immunopathogenesis [29]. In general, a Th2 immune response is favored during RSV infection, especially in younger hosts. RSV-induced pulmonary inflammation in mice was previously found to cause a shift from Th1 to Th2 cell inflammation. RSV uses multiple mechanisms to induce a Th2 cell response in the host, including RSV G protein–mediated effects [30], increasing IL-4 production from basophils [31] and induction of alternatively activated macrophages [32]. Moreover, the excessive mucus production, airway plugging, wheezing, and long-lasting effects on lung function that are common manifestations of RSV disease have some similarity with asthma, which involves a Th2-bias [29]. Therefore, strategies aimed to improve Th1 during RSV infection would beneficially modulate the outcome of the infections especially in younger hosts. In this work, we showed that nasally administered immunobiotics were able to improve respiratory Th1 response since significantly higher levels of IFN-γ were found in the respiratory tract of lactobacilli-treated mice. Then, modulation of respiratory immunity potentiated by the immunobiotic strains might contribute to an improved Th1 response and thereby favor protective immunity against viral infections such as RSV.

*c) Viability of the immunobiotics strains is not a necessary condition to achieve the immunoregulatory protective effect.* Few studies have demonstrated that the nasal administration of heat-killed immunobiotics is able to improve resistance against respiratory pathogens [11,15,16]. In this regard, earlier studies by Hori et al. [15] showed that the nasal administration of heat-killed *L. casei* Shirota stimulated cellular immunity in the respiratory tract and significantly increased the resistance of adult BALB/c mice to influenza virus infection. The authors investigated the production of various cytokines by mediastinal lymphoid node cells in mice receiving *L. casei* Shirota intranasally and found that the Shirota strain strongly induced production of IL-12 in these cells, which is an important cytokine for cytotoxic T cells and NK cells stimulation and enhancement of Th1 cytokines. In addition, both IFN-γ and TNF-α levels were improved in mediastinal lymphoid node cell cultures from mice administered *L. casei* Shirota intranasally, after influenza virus challenge [15]. Later it was reported that intranasal administration of heat-killed *L. pentosus* S-PT84 strongly enhanced Th1 immunity, IFN-α production and NK activity in the respiratory immune system and protected against influenza virus infection [33]. In addition, as mentioned above, it was demonstrated that priming of the respiratory mucosa with lactobacilli results in full protection from the otherwise lethal severe pneumovirus infection and that protection is observed in response to both live and heat-killed *L. plantarum* and *L. reuteri*[16]. That work demonstrated that nasal treatment with heat-killed immunobiotics resulted in diminished virus recovery at multiple time points and prominent suppression in the production of virus-induced proinflammatory mediators. The results of our work are in line with these previous observations since administration of both Lr05 and HkLr05 were equally effective to improve resistance of infant mice to RSV infection and reduce lung injuries. Interestingly, although both Lr06 and HkLr06 showed a similar capacity to reduce lung RSV titers, Lr06 was more effective than HkLr06 to reduce lung injuries during RSV infection. These differential effects achieved by the four treatments would be related to their specific capacities to modulate the production of IFN-γ and IL-10 after RSV challenge. All of them were able to improve respiratory IFN-γ levels and reduced viral loads, while Lr05, Lr06 and HKLr05 but not HkLr06 increased IL-10 and reduced lung injuries. Then our results suggest that not all the heat-killed bacteria derived from immunobiotic strains maintain the immunoregulatory effect after heat treatment. This would be an important point to consider when selecting immunoactive non-viable strains.

Previously, we demonstrated that the nasal treatment of malnourished mice with heat-killed *L. casei* CRL431 was able to increase their resistance to the infection with the respiratory pathogen *S. pneumoniae*[11]. The results from that study suggested that heat-killed lactobacilli are also effective in the immunomodulation of the respiratory immune system in immunocompromised hosts. Therefore, immunobiotic bacteria in the form of live cells may not be required for enhancing respiratory defenses against bacterial and viral pathogens. Our previous and present results show that non-viable immunobiotics or their cellular fractions could be an interesting alternative as mucosal adjuvants, especially in immunocompromised hosts in which the use of live bacteria might be dangerous. In addition, heat-killed immunobiotic have the advantages of allowing a longer product shelf-life, easier storage, and transportation. Therefore, to study the capacity of non-viable Lr05 or its cellular fractions to beneficially modulate the immune response against respiratory virus infections in immunocompetent and immunocompromised hosts is an interesting topic for future research.

## Conclusions

In the present work we demonstrated that nasal administration of immunobiotics is able to beneficially modulate the immune response triggered by TLR3/RIG-I activation in the respiratory tract and to increase the resistance of mice to the challenge with RSV. As it has been reported for orally administered probiotic bacteria, our results demonstrated that the immunoregulatory effect of nasally administered lactobacilli is a strain dependent effect. Comparative studies using two *Lactobacillus rhamnosus* strains of the same origin and with similar technological properties [6,7] showed that each strain has an specific immunoregulatory effect in the respiratory tract and that they differentially modulate the immune response after poly(I:C) or RSV challenges, conferring different degree of protection and using distinct immune mechanisms. We also demonstrated in this work that is possible to beneficially modulate the respiratory defenses against RSV by using heat-killed immunobiotics. Moreover, our results showed that not all heat-killed bacteria derived from viable strains with immunomodulatory capacity, are also able to functionally modulate the respiratory immune system. Therefore, detailed studies of the immunoregulatory capacities of heat-killed immunobiotics or their cellular fractions are necessary in order to find those with the highest potential to be used for improving defenses against respiratory viruses.

## Abbreviations

BAL: Broncho-alveolar lavage; BCA: Bicinchoninic acid; DCs: Dendritic cells; dsRNA: Double-stranded RNA; ELISA: Enzyme-linked immunosorbent assay; HkLr05: Heat-killed *Lactobacillus rhamnosus* CRL1505; HkLr06: Heat-killed *Lactobacillus rhamnosus* CRL1506; IL: Interleukin; IFN: Interferon; LAB: Lactic acid bacteria; LDH: Lactate dehydrogenase; Lr05: *Lactobacillus rhamnosus* CRL1505; Lr06: *Lactobacillus rhamnosus* CRL1506; NAD: Nicotinamide adenine dinucleotide; PBS: Phosphate buffer saline; RIG-I: Retinoic acid-inducible gene I; RSV: Respiratory syncytial virus; TLR3: Toll-like receptor 3; TNF: Tumor necrosis factor.

## Competing interests

The authors declare that they have no competing interests.

## Authors’ contributions

YT, EC, HZ, TT, KT and JV carried out experiments, analyzed data and performed the statistical analysis. HK, SA and JV conceived of the study, and participated in its design and coordination and helped to draft the manuscript. All authors read and approved the final manuscript.

## Funding

This study was supported by a Grant-in-Aid for Scientific Research (B)(2) (No. 21380164, 24380146) and Challenging Exploratory Research (No. 23658216) from the Japan Society for the Promotion of Science (JSPS) to Dr. H. Kitazawa and by grants from PIP 632–2009, CIUNT 26 D/403 and PICT 2010 N°1381 to Dr. Susana Alvarez and Dr. Julio Villena. J. Villena was supported by JSPS (Postdoctoral Fellowship for Foreign Researchers, Program No. 21–09335). The funders had no role in study design, data collection and analysis, decision to publish, or preparation of the manuscript.

## References

[B1] Mizgerd JP Acute lower respiratory tract infection N Engl J Med 2008 358 716 727 10.1056/NEJMra074111 18272895 PMC2711392

[B2] Falade AG Ayede AI Epidemiology, aetiology and management of childhood acute community-acquired pneumonia in developing countries–a review Afr J Med Med Sci 2011 40 293 308 22783679

[B3] Simoes EA Carbonell-Estrany X Rieger CH Mitchell I Fredrick L Groothuis JR The effect of respiratory syncytial virus on subsequent recurrent wheezing in atopic and nonatopic children J Allergy Clin Immunol 2010 126 256 262 10.1016/j.jaci.2010.05.026 20624638 PMC7126502

[B4] Weiss KA Christiaansen AF Fulton RB Meyerholz DK Varga SM Multiple CD4+ T cell subsets produce immunomodulatory IL-10 during respiratory syncytial virus infection J Immunol 2011 187 3145 3154 10.4049/jimmunol.1100764 21844390 PMC3304096

[B5] Villena J Oliveira ML Ferreira PC Salva S Alvarez S Lactic acid bacteria in the prevention of pneumococcal respiratory infection: future opportunities and challenges Int Immunopharmacol 2011 11 1633 1645 10.1016/j.intimp.2011.06.004 21708293

[B6] Salva S Villena J Alvarez S Immunomodulatory activity of Lactobacillus rhamnosus strains isolated from goat milk: Impact on intestinal and respiratory infections International Journal of Food Microbiology 2010 141 82 89 10.1016/j.ijfoodmicro.2010.03.013 20395002

[B7] Salva S Nunez M Villena J Ramon A Font G Alvarez S Development of a fermented goats' milk containing Lactobacillus rhamnosus: in vivo study of health benefits J Sci Food Agric 2011 91 2355 2362 10.1002/jsfa.4467 21604277

[B8] Villena J Racedo S Aguero G Bru E Medina M Alvarez S Lactobacillus casei improves resistance to pneumococcal respiratory infection in malnourished mice J Nutr 2005 135 1462 1469 15930453 10.1093/jn/135.6.1462

[B9] Kiyono H Fukuyama S NALT- versus Peyer's-patch-mediated mucosal immunity Nat Rev Immunol 2004 4 699 710 10.1038/nri1439 15343369 PMC7097243

[B10] Medina M Villena J Salva S Vintini E Langella P Alvarez S Nasal administration of Lactococcus lactis improves local and systemic immune responses against Streptococcus pneumoniae Microbiol Immunol 2008 52 399 409 10.1111/j.1348-0421.2008.00050.x 18667039

[B11] Villena J Barbieri N Salva S Herrera M Alvarez S Enhanced immune response to pneumococcal infection in malnourished mice nasally treated with heat-killed Lactobacillus casei Microbiol Immunol 2009 53 636 646 10.1111/j.1348-0421.2009.00171.x 19903264

[B12] Villena J Chiba E Tomosada Y Salva S Marranzino G Kitazawa H Alvarez S Orally administered Lactobacillus rhamnosus modulates the respiratory immune response triggered by the viral pathogen-associated molecular pattern poly(I:C) BMC Immunol 2012 13 53 10.1186/1471-2172-13-53 22989047 PMC3460727

[B13] Londhe VA Belperio JA Keane MP Burdick MD Xue YY Strieter RM CXCR2 is critical for dsRNA-induced lung injury: relevance to viral lung infection J Inflamm (Lond) 2005 2 4 10.1186/1476-9255-2-4 15921526 PMC1156932

[B14] Stowell NC Seideman J Raymond HA Smalley KA Lamb RJ Egenolf DD Bugelski PJ Murray LA Marsters PA Bunting RA Long-term activation of TLR3 by poly(I:C) induces inflammation and impairs lung function in mice Respir Res 2009 10 43 10.1186/1465-9921-10-43 19486528 PMC2694181

[B15] Hori T Kiyoshima J Shida K Yasui H Effect of intranasal administration of Lactobacillus casei Shirota on influenza virus infection of upper respiratory tract in mice Clin Diagn Lab Immunol 2001 8 593 597 11329464 10.1128/CDLI.8.3.593-597.2001 PMC96107

[B16] Gabryszewski SJ Bachar O Dyer KD Percopo CM Killoran KE Domachowske JB Rosenberg HF Lactobacillus-mediated priming of the respiratory mucosa protects against lethal pneumovirus infection J Immunol 2011 186 1151 1161 10.4049/jimmunol.1001751 21169550 PMC3404433

[B17] Barbieri N Villena J Herrera M Salva S Alvarez S Nasally administered Lactobacillus rhamnosus accelerate the recovery of humoral immunity in B lymphocyte-deficient malnourished mice J Nutr 2013 143 227 235 10.3945/jn.112.165811 23269656

[B18] Murawski MR Bowen GN Cerny AM Anderson LJ Haynes LM Tripp RA Kurt-Jones EA Finberg RW Respiratory syncytial virus activates innate immunity through Toll-like receptor 2 J Virol 2009 83 1492 1500 10.1128/JVI.00671-08 19019963 PMC2620898

[B19] Hori T Kiyoshima J Shida K Yasui H Augmentation of cellular immunity and reduction of influenza virus titer in aged mice fed Lactobacillus casei strain Shirota Clin Diagn Lab Immunol 2002 9 105 108 11777838 10.1128/CDLI.9.1.105-108.2002 PMC119906

[B20] Sha Q Truong-Tran AQ Plitt JR Beck LA Schleimer RP Activation of airway epithelial cells by toll-like receptor agonists Am J Respir Cell Mol Biol 2004 31 358 364 10.1165/rcmb.2003-0388OC 15191912

[B21] Furuhashi K Suda T Hasegawa H Suzuki Y Hashimoto D Enomoto N Fujisawa T Nakamura Y Inui N Shibata K Mouse lung CD103+ and CD11bhigh dendritic cells preferentially induce distinct CD4+ T-cell responses Am J Respir Cell Mol Biol 2012 46 165 172 10.1165/rcmb.2011-0070OC 21908266

[B22] Yoda K Miyazawa K Harata G He F Kitazawa H, Villena J, Alvarez S Immunobiotics and antiviral immunity Immunobiotics and Immunogenics 2013 Enfield, New Hampshire, USA: Science Publishers CRC Press Taylor & Francis Group

[B23] Harata G He F Hiruta N Kawase M Kubota A Hiramatsu M Yausi H Intranasal administration of Lactobacillus rhamnosus GG protects mice from H1N1 influenza virus infection by regulating respiratory immune responses Lett Appl Microbiol 2010 50 597 602 10.1111/j.1472-765X.2010.02844.x 20438620

[B24] Li F Zhu H Sun R Wei H Tian Z Natural killer cells are involved in acute lung immune injury caused by respiratory syncytial virus infection J Virol 2012 86 2251 2258 10.1128/JVI.06209-11 22171263 PMC3302418

[B25] Bem RA Domachowske JB Rosenberg HF Animal models of human respiratory syncytial virus disease Am J Physiol Lung Cell Mol Physiol 2011 301 L148 L156 10.1152/ajplung.00065.2011 21571908 PMC3154630

[B26] Rutigliano JA Graham BS Prolonged production of TNF-alpha exacerbates illness during respiratory syncytial virus infection J Immunol 2004 173 3408 3417 15322205 10.4049/jimmunol.173.5.3408

[B27] Loebbermann J Schnoeller C Thornton H Durant L Sweeney NP Schuijs M O'Garra A Johansson C Openshaw PJ IL-10 regulates viral lung immunopathology during acute respiratory syncytial virus infection in mice PLoS One 2012 7 e32371 10.1371/journal.pone.0032371 22393401 PMC3290561

[B28] McNamara PS Smyth RL The pathogenesis of respiratory syncytial virus disease in childhood Br Med Bull 2002 61 13 28 10.1093/bmb/61.1.13 11997296

[B29] Collins PL Melero JA Progress in understanding and controlling respiratory syncytial virus: still crazy after all these years Virus Res 2011 162 80 99 10.1016/j.virusres.2011.09.020 21963675 PMC3221877

[B30] Graham BS Johnson TR Peebles RS Immune-mediated disease pathogenesis in respiratory syncytial virus infection Immunopharmacology 2000 48 237 247 10.1016/S0162-3109(00)00233-2 10960663

[B31] Moore ML Newcomb DC Parekh VV Van Kaer L Collins RD Zhou W Goleniewska K Chi MH Mitchell D Boyce JA STAT1 negatively regulates lung basophil IL-4 expression induced by respiratory syncytial virus infection J Immunol 2009 183 2016 2026 10.4049/jimmunol.0803167 19587017 PMC3755459

[B32] Shirey KA Pletneva LM Puche AC Keegan AD Prince GA Blanco JC Vogel SN Control of RSV-induced lung injury by alternatively activated macrophages is IL-4R alpha-, TLR4-, and IFN-beta-dependent Mucosal Immunol 2010 3 291 300 10.1038/mi.2010.6 20404812 PMC2875872

[B33] Izumo T Maekawa T Ida M Noguchi A Kitagawa Y Shibata H Yasui H Kiso Y Effect of intranasal administration of Lactobacillus pentosus S-PT84 on influenza virus infection in mice Int Immunopharmacol 2010 10 1101 1106 10.1016/j.intimp.2010.06.012 20601181

